# Vastus medialis cross-sectional area is positively associated with patella cartilage and bone volumes in a pain-free community-based population

**DOI:** 10.1186/ar2573

**Published:** 2008-12-15

**Authors:** Patricia A Berry, Andrew J Teichtahl, Ana Galevska-Dimitrovska, Fahad S Hanna, Anita E Wluka, Yuanyuan Wang, Donna M Urquhart, Dallas R English, Graham G Giles, Flavia M Cicuttini

**Affiliations:** 1 Department of Epidemiology and Preventive Medicine, Monash University Central and Eastern Clinical School, Commercial Road, Melbourne, 3004, Australia; 2 Baker Heart Research Institute, Commercial Road, Melbourne, 3004, Australia; 3 Cancer Epidemiology Centre, The Cancer Council of Victoria, Rathdowne Street, Melbourne, 3053, Australia; 4 School of Population Health, The University of Melbourne, Bouverie Street, Melbourne, 3010, Australia

## Abstract

**Introduction:**

Although vastus medialis and lateralis are important determinants of patellofemoral joint function, their relationship with patellofemoral joint structure is unknown. The aim of this study was to examine potential determinants of vastus medialis and lateralis cross-sectional areas and the relationship between the cross-sectional area and patella cartilage and bone volumes.

**Methods:**

Two hundred ninety-seven healthy adult subjects had magnetic resonance imaging of their dominant knee. Vastus medialis and lateralis cross-sectional areas were measured 37.5 mm superior to the quadriceps tendon insertion at the proximal pole of the patella. Patella cartilage and bone volumes were measured from these images. Demographic data and participation in vigorous physical activity were assessed by questionnaire.

**Results:**

The determinants of increased vastus medialis and lateralis cross-sectional areas were older age (*P *≤ 0.002), male gender (*P *< 0.001), and greater body mass index (*P *≤ 0.07). Participation in vigorous physical activity was positively associated with vastus medialis cross-sectional area (regression coefficient [beta] 90.0; 95% confidence interval [CI] 38.2, 141.7) (*P *< 0.001) but not with vastus lateralis cross-sectional area (beta 10.1; 95% CI -18.1, 38.3) (*P *= 0.48). The cross-sectional area of vastus medialis only was positively associated with patella cartilage volume (beta 0.6; 95% CI 0.23, 0.94) (*P *= 0.001) and bone volume (beta 3.0; 95% CI 1.40, 4.68) (*P *< 0.001) after adjustment for potential confounders.

**Conclusions:**

Our results in a pain-free community-based population suggest that increased cross-sectional area of vastus medialis, which is associated with vigorous physical activity, and increased patella cartilage and bone volumes may benefit patellofemoral joint health and reduce the long-term risk of patellofemoral pathology.

## Introduction

Vastus medialis and lateralis are recognised as important determinants of patellofemoral joint function. In particular, these muscles help prevent excessive patella displacement and contribute to patellofemoral joint congruency and stability [1]. Deficiencies in neuromotor control, such as delayed activation of vastus medialis, have been linked to patellofemoral pain syndrome [2] and are speculated to contribute to the risk of patellofemoral subluxation/dislocation [3].

Muscle cross-sectional area, which can be reliably measured via magnetic resonance imaging (MRI) [4] or computerised tomography [5], is representative of the force-producing capability of a muscle [6]. Previous studies have examined the cross-sectional area of the quadriceps and have used the mid-thigh level to assess the maximum force-producing capability of the quadriceps muscle group [5,7,8]. Studies examining spinal pathology have demonstrated that reduced cross-sectional area of local muscles that are in close proximity to the joint, attaching directly to the lumbar spine, are associated with low back pain, whereas increased cross-sectional area of these muscles contributes to spinal control [9-11]. However, it is unclear whether an increased cross-sectional area of local muscles at other anatomical sites confers similar benefits. Although distal vastus medialis and lateralis muscles have been implicated in patellofemoral pain [2] and subluxation/dislocation [3], their association with patellofemoral joint structures is unclear. Similarly, the determinants of vastus medialis and lateralis cross-sectional areas are unclear.

In a community-based population of women, we recently showed that increased vastus medialis cross-sectional area was positively associated with patella bone volume, but not patella cartilage volume [12]. Whether this relationship is similar in males is unknown. Moreover, whether the lack of a relationship with cartilage was a true effect or was due to inadequate power of that study is unknown. Thus, the aim of this study was to examine the determinants of vastus medialis and lateralis cross-sectional areas and whether vastus medialis cross-sectional area is associated with patella cartilage volume. In addition, we aimed to determine whether these findings are similar in males and females.

## Materials and methods

### Subjects

Subjects were recruited from an existing cohort – the Melbourne Collaborative Cohort Study (MCCS), a prospective cohort study of community-based people 40 to 69 years old at recruitment (1990 to 1994) – with the aim of examining the role of lifestyle and genetic factors in the risk of cancer and chronic diseases from middle age and beyond, as previously described [13]. Individuals were excluded if in the last 5 years they had knee pain lasting for more than 24 hours, a previous knee injury requiring non-weight-bearing treatment for more than 24 hours or surgery (including arthroscopy), or a history of any arthritis diagnosed by a medical practitioner. A further exclusion criterion was a contraindication to MRI. The study was approved by The Cancer Council Victoria's Human Research Ethics Committee and the Standing Committee on Ethics in Research Involving Humans of Monash University (Melbourne, Australia). All participants gave written informed consent.

### Data collection

Height and weight were measured and body mass index (BMI) was calculated. At MCCS recruitment from 1990 to 1994, subjects completed a questionnaire that collected demographic and physical activity information. Subjects were asked, 'On average (over the last 6 months), how many times a week did you exercise vigorously for a period of 20 minutes?' Frequency of weekly episodes of vigorous physical activity was categorised into three groups: never, once or twice per week, and at least three times per week. Subjects were categorised as participating in vigorous physical activity if they exercised once or twice per week or at least three times per week. Vigorous was defined as activity leading to sweating or dyspnoea, and examples such as swimming, tennis, netball, athletics (which may involve running, walking, throwing or jumping), and running were listed.

### Magnetic resonance imaging

MRI was performed on the dominant knee as previously described [14]. The following sequence and parameters were used: a T1-weighted fat-suppressed three-dimensional gradient recall acquisition in the steady state; flip angle 55 degrees; repetition time 58 milliseconds; echo time 12 milliseconds; field of view 16 cm; 60 partitions; 512 (frequency direction, superior-inferior) × 512 (phase-encoding direction, anterior-posterior) matrix; one acquisition, time 11 minutes 56 seconds. Sagittal images were obtained at a partition thickness of 1.5 mm and an in-plane resolution of 0.31 mm × 0.31 mm (512 × 512 pixels). The sagittal MR images were reformatted in the axial plane with a partition thickness of 1.25 mm and an in-plane resolution of 0.31 × 0.31 mm (512 × 512 pixels).

### Vastus medialis and lateralis cross-sectional areas

Distal vastus medialis and lateralis cross-sectional areas were measured directly from axial images by one trained observer manually drawing disarticulation contours around the muscle boundaries using the independent workstation software Osiris (Digital Imaging Unit, University Hospital of Geneva, Geneva, Switzerland). The cross-sectional area was measured at the MR slice 37.5 mm superior to the quadriceps tendon insertion at the proximal pole of the patella, orthogonal to the long axis of the leg. This slice was chosen as it was the largest slice visible across all subjects. The intraobserver reliability for vastus muscle cross-sectional area (expressed as intraclass correlation coefficient) was 0.99.

### Measurement of patella cartilage and bone volumes

Patella cartilage and bone volumes were determined by manually drawing disarticulation contours around the patella boundaries on images 1.5 mm apart on sagittal views, using image processing on an independent workstation using the Osiris software as previously described [14,15]. The coefficients of variation were 2.1% for patella cartilage volume and 2.2% for patella bone volume [16].

### Statistical analyses

Outcome variables, including distal vastus medialis and lateralis cross-sectional areas and cartilage and bone volumes, were initially assessed for normality and were found to approximate the normal distribution. Univariable and multiple linear regression models were used to examine determinants of vastus medialis and lateralis cross-sectional areas and the relationship with patella cartilage and bone volumes. Potential confounders, including age, gender, BMI, and participation in vigorous physical activity, were adjusted for in multivariate analyses. *R*^2 ^values were calculated to determine the proportion of variance explained by the multiple regression equation. All analyses were performed using the SPSS statistical package (standard version 14.0; SPSS Inc., Chicago, IL, USA). A *P *value of less than 0.05 was considered statistically significant.

## Results

The characteristics of the 297 subjects (63% women) who participated in the study are presented in Table 1.

**Table 1:** Characteristics of the study population (n = 297)

	Mean (SD)^a^
Age at magnetic resonance imaging, years	59.1 (6.3)
Females, number (percentage)	186 (63)
Body mass index, kg/m^2^	25.2 (3.8)
Participation in vigorous physical activity, number of subjects (percentage)^b^	107 (36)
Distal vastus medialis cross-sectional area, mm^2^	1,171 (306)
Distal vastus lateralis cross-sectional area, mm^2^	333 (127)
Patella cartilage volume, mm^3^	2,656 (886)
Patella bone volume, mm^3^	20,276 (4,667)

^a^Values are reported as mean (standard deviation, SD) unless otherwise stated. ^b^Participation in vigorous physical activity at least once or twice a week or at least three times per week (over the last 6 months) for a period of 20 minutes that leads to sweating or dyspnoea.

### Determinants of distal vastus medialis and lateralis muscle cross-sectional areas

The determinants of vastus medialis and lateralis cross-sectional areas are presented in Table 2. After adjustment for potential confounders, including gender, BMI, and participation in vigorous physical activity, we found that age was negatively associated with vastus medialis and with vastus lateralis cross-sectional area. Women had significantly smaller vastus medialis and vastus lateralis cross-sectional area than men in multivariate analysis. BMI was positively associated with vastus medialis cross-sectional area in multivariate analysis, with a similar trend observed with vastus lateralis. Participation in vigorous physical activity (binary variable) was positively associated with vastus medialis cross-sectional area in multivariate analysis, whereas no significant association was seen for vastus lateralis. In multivariate analysis, similar results were obtained when the relationship between increasing frequency of weekly episodes of vigorous physical activity and vastus medialis (beta 54.9; 95% confidence interval [CI] 24.6, 85.2) (*P *= 0.0004) and vastus lateralis (beta 3.0; 95% CI -13.6, 19.5) (*P *= 0.73) was considered.

**Table 2:** Determinants of distal vastus medialis and vastus lateralis cross-sectional areas

	Univariate regression coefficient (95% CI)	Multivariate regression coefficient (95% CI)^a^	*R*^2 ^for model^a^
Vastus medialis cross-sectional area, mm^2^			
Age, years	-1.39 (-7.78, 4.99)	-7.1 (-11.7, -2.6)^b^	0.532
Gender	-415.9 (-470.2, -361.5)^b^	-391.6 (-443.0, -340.2)^b^	0.532
Body mass index, kg/m^2^	27.5 (18.9, 36.2)^b^	21.7 (15.2, 28.2)^b^	0.532
Participation in vigorous physical activity, yes/no	118.6 (47.0, 190.1)^b^	90.0 (38.2, 141.7)^b^	0.532
Vastus lateralis cross-sectional area, mm^2^			
Age, years	-1.7 (-4.4, 0.94)	-3.3 (-5.8, -0.8)^b^	0.192
Gender	-105.6 (-133.0, -78.2)^b^	-106.0 (-134.0, -78.0)^b^	0.192
Body mass index, kg/m^2^	4.8 (1.1, 8.6)^c^	3.3 (-0.2, 6.8)	0.192
Participation in vigorous physical activity, yes/no	23.2 (-6.8, 53.3)	10.1 (-18.1, 38.3)	0.192

^a^Age, gender, body mass index, and participation in vigorous physical activity included in multivariate regression equation. ^b^*P *< 0.01. ^c^*P *< 0.05. CI, confidence interval.

### Relationship between distal vastus medialis and lateralis cross-sectional areas and patella structures

The association between vastus medialis and lateralis cross-sectional areas and patella structures, including cartilage and bone volumes, is presented in Table 3. Vastus medialis, but not vastus lateralis, cross-sectional area was positively associated with patella cartilage volume and bone volume in multivariate analysis. To determine whether gender differences in effect exist, men and women were analysed separately in multivariate analyses. The relationship between vastus medialis cross-sectional area and patella cartilage volume was similar for men (beta 0.68; 95% CI -0.01, 1.38) (*P *= 0.054) and women (beta 0.38; 95% CI -0.02, 0.77) (*P *= 0.06), and the relationship between vastus medialis cross-sectional area and patella bone volume was also similar for men (beta 3.79; 95% CI 0.95, 6.63) (*P *= 0.01) and women (beta 2.16; 95% CI 0.07, 4.24) (*P *= 0.04). Similar results were obtained when height and weight were used in the multivariate regression equation instead of BMI (data not shown).

**Table 3:** Relationship between distal vastus medialis and vastus lateralis cross-sectional areas and patella structures

	Univariate regression coefficient (95% CI)	Multivariate regression coefficient (95% CI)	*R*^2 ^for model
Vastus medialis cross-sectional area, mm^2^			
Patella cartilage volume, mm^3^	1.5 (1.3, 1.8)^a^	0.58 (0.23, 0.94)^a^	0.472^b^
Patella bone volume, mm^3^	9.09 (7.69, 10.50)^a^	3.04 (1.40, 4.68)^a^	0.592^c^
Vastus lateralis cross-sectional area, mm^2^			
Patella cartilage volume, mm^3^	1.67 (0.89, 2.44)^a^	-0.33 (-0.99, 0.33)	0.455^b^
Patella bone volume, mm^3^	10.3 (6.19, 14.31)^a^	-1.31 (-4.40, 1.78)	0.574^c^

^a^*P *< 0.01. ^b^Age, gender, body mass index, and participation in vigorous physical activity and patella cartilage volume included in the multivariate regression equation. ^c^Age, gender, body mass index, and participation in vigorous physical activity and patella bone volume included in the multivariate regression equation. CI, confidence interval.

## Discussion

Our results have demonstrated that older age, female gender, and lower BMI are associated with reduced vastus medialis and lateralis cross-sectional areas. Participation in vigorous physical activity was associated with increased vastus medialis, but not vastus lateralis, cross-sectional area. Similarly, only increased vastus medialis cross-sectional area was associated with increased patella cartilage and bone volumes. These findings suggest that increased vastus medialis cross-sectional area may benefit patellofemoral joint health.

Our measure of vastus medialis and lateralis cross-sectional areas demonstrated expected relationships, with age and female gender being associated with a reduction in vastus medialis and lateralis cross-sectional areas, whereas BMI was associated with greater vastus medialis and lateralis cross-sectional areas. Though not examined specifically in this study, a reduction in muscle size has been shown to be common with aging [4,17,18], and it has been shown that females have, on average, less muscle mass relative to males [17,19]. In contrast, a larger BMI may be inherently associated with increased muscle cross-sectional area due to a larger body size. We have also demonstrated that participation in vigorous physical activity was associated with increased vastus medialis, but not vastus lateralis, cross-sectional area. A primary action of the distal portion of vastus medialis is to dynamically restrain the natural tendency of the patella to track laterally [1]. Therefore, the relationship between vastus medialis cross-sectional area and participation in physical activity may, in the setting of vigorous physical activity, reflect vastus medialis hypertrophy in an attempt to restrain excessive lateral patella displacement.

We have previously examined the relationship between vastus medialis and lateralis cross-sectional areas and patella structures [12] in an independent and smaller (n = 175) cohort of healthy women (mean age of 52 years). In this previous study, we demonstrated a positive association between vastus medialis cross-sectional area and patella bone volume, but not cartilage volume. In the present study, we examined an entirely independent cohort and substantiated all previously significant findings while also noting that increased patella cartilage volume is significantly associated with an increased cross-sectional area of vastus medialis. The discrepancy in cartilage volume results between the two independent studies may be attributable to the smaller sample size of our previous study. Moreover, in the present study, we included males and thus expanded the generalisability of our findings. Subsequently, the present study substantiates previous findings pertaining to both men and women. In the past and present studies, we have found vastus medialis, rather than vastus lateralis, to be the significant determinant of patella structures. The relative importance of vastus medialis at the patellofemoral joint is also supported by electromyography studies, which demonstrated that delayed activation of vastus medialis relative to vastus lateralis is associated with patellofemoral pathologies, including pain [2] and subluxation/dislocation [3].

How a greater vastus medialis cross-sectional area mediates increased patella cartilage and bone volumes is unclear. Previous studies have shown that reduced cross-sectional areas of local spinal muscles are associated with instability and low back pain [9-11]. As indicated by extrapolating such data to the patellofemoral joint, it may be that a greater cross-sectional area of the vastus medialis muscle helps to prevent the natural tendency of the patella to track laterally [1] and thus reduce any shearing damage that may occur to articular surfaces. This, in turn, may produce an optimal biomechanical environment and have a beneficial effect on patella structures, including cartilage and bone. Moreover, our recent longitudinal data have suggested that increased patella bone volume may be advantageous to patellofemoral joint health, as increased baseline bone volume was associated with a reduction in the rate of patella cartilage volume loss [20]. Therefore, it may be that increased vastus medialis cross-sectional area benefits the patellofemoral joint via biomechanical effects on both patella bone and cartilage volumes. Longitudinal studies are required to determine whether increased vastus medialis cross-sectional area reduces the risk of long-term patellofemoral pathology.

We examined a healthy population without knee pain or pathology and our results cannot be generalised to symptomatic populations or those with established knee pathology. Nevertheless, studying a healthy population allows the identification of factors that may be associated with early structural changes at the knee and reduces the confounding effect of reduction in muscle size due to pain-related disuse. Furthermore, this study may have been limited by our method for assessing the cross-sectional area of the distal vastus medialis and lateralis muscles. We measured the vastus muscles at the MR slice 37.5 mm superior to the quadriceps tendon insertion at the proximal pole of the patella. This slice was chosen as it was the largest slice visible across all subjects. Moreover, we have calculated cross-sectional area rather than the physiological cross-sectional area (PCSA) that previously has been employed to investigate muscle-force relationships [21]. Calculating the PCSA requires imaging of the entire length of the muscle fibre, which would be a costly and timely exercise using MRI. However, our measure of cross-sectional area showed the expected relationships with age, gender, and BMI. It may be hypothesised that people with larger muscles inherently have larger joint structures, including cartilage and bone volumes, but if this were the case, vastus lateralis would have been significantly associated with patella cartilage and bone. Moreover, the relationships observed in this study were independent of BMI, height, and weight (data not shown) as a measure of body size. Although the determinants of patella structure remain unclear, there is likely to be a complex interplay between the surrounding soft tissue support (for example, vastus medialis and lateralis muscles) and the bony geometry (for example, femoral sulcus angle and patella tilt). Therefore, future studies may benefit from examining other articular supports that may also contribute to patellofemoral joint structure and function.

## Conclusion

Our results in a pain-free population without clinical knee osteoarthritis suggest that increased cross-sectional area of vastus medialis, which is associated with participation in vigorous physical activity, and increased patella cartilage and bone volumes, may benefit patellofemoral joint health. This warrants further investigation as a potential method for reducing long-term patellofemoral pathology.

## Abbreviations

BMI: body mass index; CI: confidence interval; MCCS: Melbourne Collaborative Cohort Study; MR: magnetic resonance; MRI: magnetic resonance imaging; PCSA: physiological cross-sectional area.

## Competing interests

The authors declare that they have no competing interests.

## Authors' contributions

FMC and AEW participated in the design of the study and in the analysis and interpretation of data and reviewed the manuscript. DRE and GGG participated in the acquisition of data and in the analysis and interpretation of data and reviewed the manuscript. FSH participated in carrying out the measurement of muscle structure and in the analysis and interpretation of data and reviewed the manuscript. AG-D participated in carrying out the measurement of muscle structure. YW carried out the measurement of knee cartilage and bone structure, participated in the analysis and interpretation of data, and reviewed the manuscript. PAB and AJT performed the statistical analysis and interpretation of data and drafted the manuscript. DMU participated in the analysis and interpretation of data and reviewed the manuscript. All authors read and approved the final manuscript.
